# A 1D imaging soft X-ray spectrometer for the small quantum systems instrument at the European XFEL

**DOI:** 10.1107/S1600577524005988

**Published:** 2024-07-30

**Authors:** Marcus Agåker, Johan Söderström, Thomas M. Baumann, Carl-Johan Englund, Ludvig Kjellsson, Rebecca Boll, Alberto De Fanis, Simon Dold, Tommaso Mazza, Jacobo Montaño, Astrid Münnich, Terence Mullins, Yevheniy Ovcharenko, Nils Rennhack, Philipp Schmidt, Björn Senfftleben, Monica Turcato, Sergey Usenko, Michael Meyer, Joseph Nordgren, Jan-Erik Rubensson

**Affiliations:** a Department of Physics and Astronomy, Uppsala University, Box 516, 751 20Uppsala, Sweden; b MAX IV Laboratory, Lund University, Box 118, 221 00Lund, Sweden; c European XFEL, Holzkoppel 4, 22869Schenefeld, Germany; RIKEN SPring-8 Center, Japan

**Keywords:** soft X-ray scattering, resonant inelastic X-ray scattering, RIXS, soft X-ray spectrometers, X-ray free-electron lasers, XFEL

## Abstract

A 1D imaging soft X-ray spectrometer installed on the small quantum systems (SQS) scientific instrument of the European XFEL is described. It uses movable cylindrical constant-line-spacing gratings in the Rowland configuration for energy dispersion in the vertical plane, and Wolter optics for simultaneous 1D imaging of the source in the horizontal plane. The soft X-ray fluorescence spectro-imaging capability will be exploited in pump–probe measurements and in investigations of propagation effects and other nonlinear phenomena.

## Abstract 2

A 1D imaging soft X-ray spectrometer, installed and commissioned on the SQS instrument of the European XFEL, is described.

## 1. Introduction

Soft X-ray emission spectroscopy (XES) studies transitions where a valence electron fills a core vacancy. The process maps the electronic structure of the system with atomic site selectivity, and due to the short core–hole lifetime the spectra also provide information about the excitation–emission dynamics. When undulators were inserted into synchrotrons in the mid-1980s, the brilliance of the radiation facilitated measurements of XES spectra, selectively excited with tunable monochromated X-rays (Nordgren *et al.*, 1989 ▸). The description of the underlying process, in terms of two separate steps of selective excitation followed by emission, is often insufficient. It must rather be treated as a second-order resonant inelastic X-ray scattering (RIXS) process. This has far-reaching consequences. As fundamental conservation laws apply to the scattering event, energy and momentum transfer can be determined with high precision, enabling detailed investigation of elementary excitations. With refined instrumentation and new generations of photon sources, RIXS has now evolved into a powerful method with scientific applications ranging from fundamental molecular systems (Gel’mukhanov *et al.*, 2021 ▸) to materials with strong electron correlation (Ament *et al.*, 2011 ▸; Gilmore *et al.*, 2021 ▸).

X-ray free-electron lasers (XFELs) provide high-intensity X-ray pulses of short duration, opening a new window of opportunity for investigation of ultra-fast phenomena by exploiting pump–probe schemes and nonlinear X-ray physics. It can be anticipated that high-resolution XES will be an important tool when investigating fine details in the inter­action dynamics. An advantage of photon-in–photon-out experiments at high intensity is that the signal is unaffected by plasma potentials and high fields, which may distort the signal in experiments where charged particles are monitored. For time-resolved studies, a process is typically initiated by an optical laser pulse, and the evolution of this process is probed by the ordinary RIXS process (Dell’Angela *et al.*, 2013 ▸; Wernet *et al.*, 2015 ▸). With sufficient time resolution, access to coherent electronic vibronic dynamics in molecules is within reach. Many fascinating nonlinear X-ray phenomena are predicted (Sun *et al.*, 2010 ▸; Biggs *et al.*, 2012 ▸; Li *et al.*, 2020 ▸), with stimulated RIXS (Weninger *et al.*, 2013 ▸) being a fundamental building block (Kimberg *et al.*, 2016 ▸). The vision is to enable the extension of the numerous spectroscopic methods using optical lasers for control and manipulation of quantum dynamics to the X-ray range.

**Figure 1:**
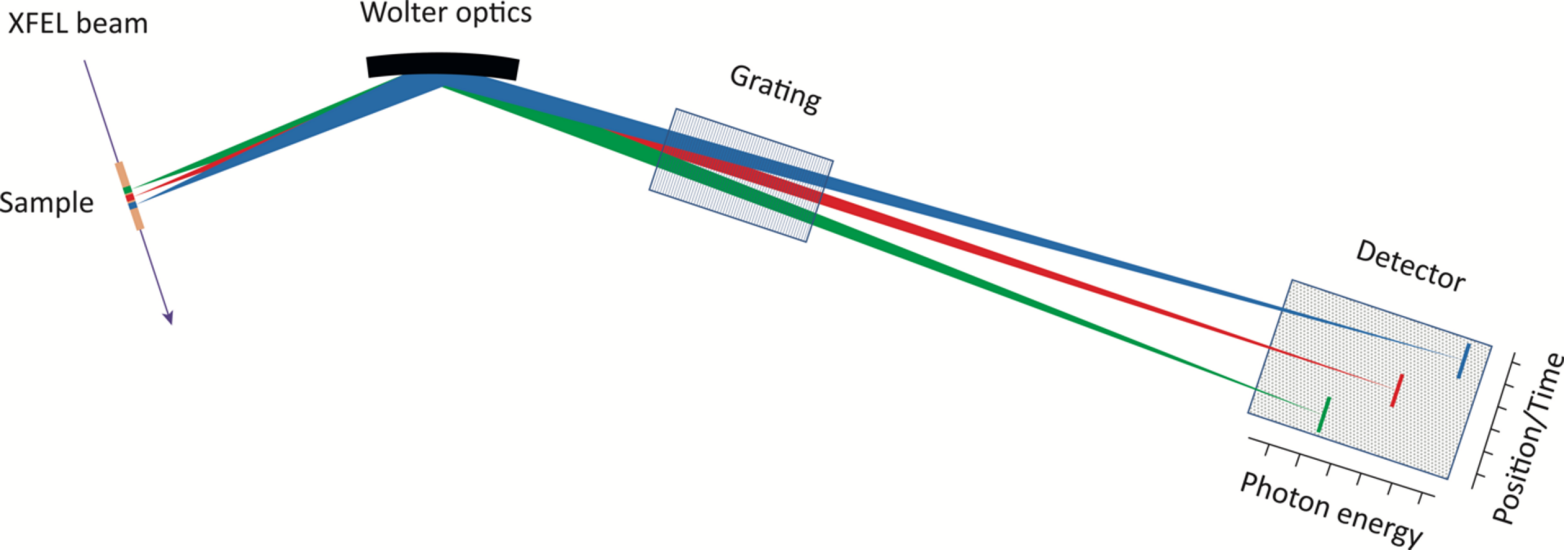
A top view showing the principle of the 1D imaging soft X-ray spectrometer. A Wolter mirror pair forms a magnified image of the sample along the XFEL beam on the detector in the horizontal plane, while the grating diffraction forms a wavelength-dispersed spectrum in the perpendicular plane. The detector image shows spectral features unique to the three particular positions indicated in the sample along the XFEL beam.

To facilitate this development a novel spectrometer has been installed on the small quantum systems (SQS) instrument (Tschentscher *et al.*, 2017 ▸; Mazza *et al.*, 2023 ▸; https://www.xfel.eu/facility/instruments/sqs/index_eng.html) at the Euro­pean XFEL (EuXFEL) (Decking *et al.*, 2020 ▸). The spectrometer records high-energy-resolution X-ray emission spectra perpendicular to the propagation direction of the incident XFEL beam. At the same time it has 1D imaging capability that allows for separation of emission produced at various positions along the path of the propagating incident beam (Figs. 1 ▸ and 2 ▸). This implies that the output from the spectrometer can be separated into emission spectra recorded at various positions along the XFEL beam. The imaging capability serves several purposes.

First, the properties of the incoming pulse can change as it propagates in dense gaseous media, thereby changing the probability of exciting atoms or molecules along its path. In the linear regime, we expect an attenuation of the signal according to the Beer–Lambert law. As soon as nonlinear processes become important the behavior becomes highly non-trivial, and any intensity variation along the beam may lead to a variation in the emission spectrum. Predicted nonlinear X-ray optical effects include substantial reshaping and slowing down of the pulse as it proceeds through the medium (Sun *et al.*, 2010 ▸; Li *et al.*, 2020 ▸). In addition, stimulated emission is enhanced along the path (Weninger *et al.*, 2013 ▸) and this development can be monitored via the X-ray fluorescence in the perpendicular direction. The high spatial resolution along the incident beam will improve the understanding of these nonlinear phenomena and facilitate the development of associated emerging methodologies.

**Figure 2:**
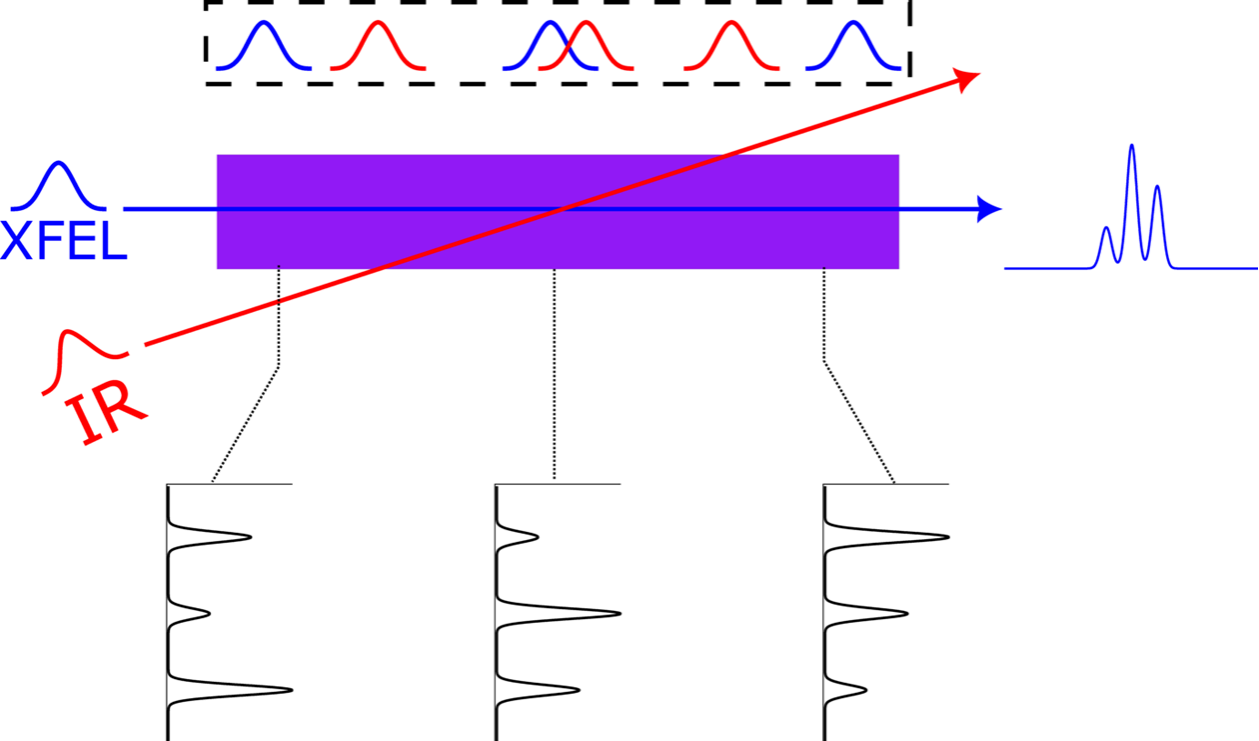
An illustration of how the spectrometer is exploited for pump–probe measurements. A laser beam (red) enters nearly collinearly with the XFEL beam (blue). Due to the small angle between the two rays, the effective propagation time of the pulses differs. Any spectral variation as a function of the delay is monitored in the 1D spectrometer. The spatial resolution along the incident beam corresponds to a few femtoseconds time resolution of the delay. The box in the top part of the figure schematically shows the pulses and their temporal delay along the propagation of the XFEL beam at different points along the interaction medium. Spectra recorded at three different positions, corresponding to three different delays, are shown in the bottom part of the figure. Typically, the measurements by the 1D spectrometer are combined with measurements by an in-line spectrometer, which directly monitors transient absorption and stimulated emission, indicated to the right in the figure.

Second, the spatial resolution can be exploited in pump–probe experiments. The experimental chamber is prepared for in-coupling of an optical laser at a small angle compared to the XFEL beam, so that the effective delay between the SASE and optical laser pulses varies in the field of view of the spectrometer (Fig. 2 ▸). Assuming that both pulses propagate at the speed of light in a vacuum, variations due to very small (sub-femtosecond) resolvable delays can be monitored with the spectrometer. Although this does not eliminate the global jitter between pulses (Rivas *et al.*, 2022 ▸), it will be a valuable asset in optical laser pump–X-ray probe experiments. In dense media a collinear laser in-coupling can also be conceived, where the delay variations due to the different group velocities of the two pulses are exploited. The latter scheme can also be used in two-color X-ray pump–probe experiments, where the propagation speed can be substantially different if the photon energy is tuned off or on resonances (Sun *et al.*, 2010 ▸; Li *et al.*, 2020 ▸).

The temporal resolution in pump–probe measurements will be defined by several factors. Firstly, the pulse duration of the two (or more) pulses will affect the overall temporal resolution, as is common in pump–probe measurements. Secondly, if the photon energies are significantly separated, or the first pulse alters the sample composition (*e.g.* creates a plasma), the refractive indices for the two pulses can be significantly different, which would lead to temporal walk-off between the pulses. In many cases such effects can be treated in the analysis, but it is important to be aware of them. Thirdly, in the case of pulses with a non-collinear propagation direction as shown schematically in Fig. 2 ▸, the temporal resolution will furthermore be affected by the angle between the two pulses. In such experiments the temporal resolution will be defined by the origin of the emission, as shown schematically in Fig. 2 ▸, and thus a delay stage is not needed. Assuming that the two pulses both travel at the same velocity (*c*_0_) and that they are infinitely short in time, the temporal resolution is given by the angle between the two Poynting vectors and by the spatial dimension imaged by the spectrometer (2 mm). Assuming a spatial resolution of 15 µm over the whole 2 mm imaged by the spectrometer, the temporal resolution will be defined by
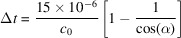
and the maximum temporal delay is defined by
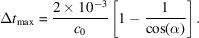
While the angle α can in theory be freely tunable, the setup easily allows for an angle of α = 15°. This corresponds to 1.8 fs between each 15 µm segment and a maximum total delay of 0.24 ps. This will be further impacted by the previously discussed temporal broadenings and by pulse-to-pulse jitter.

In general, the dynamics of transient high-energy-density plasmas and highly charged ions, formed by intense XFEL pulses (Ren *et al.*, 2023 ▸), can be investigated using the new imaging spectrometer. This may have ramifications in all sub-fields of plasma physics, including astrophysics and fusion research. In addition, it allows XES of transient species, like multiply charged molecules, that are otherwise not accessible. Finally, it has recently been demonstrated that X-ray fluorescence can be exploited in the imaging of un-crystallized macromolecules (Trost *et al.*, 2023 ▸) with XFEL pulses. We foresee that the spectrometer described here will also be useful in the refinement of such methods.

The SQS instrument at the EuXFEL covers the energy range 270–3000 eV, where the XFEL can provide SASE pulses with an energy of up to 10 mJ and a typical duration of 25 fs (down to sub-femtosecond with reduced energy in special operation) in a beam focused to micrometre size (Mazza *et al.*, 2023 ▸; Baumann *et al.*, 2023 ▸). This is sufficient for exploitation of the nonlinear phenomena described above, and the high-repetition rate of the EuXFEL facilitates fast data collection. The two-color X-ray mode (Serkez *et al.*, 2020 ▸) is routinely provided, and a synchronized optical laser is also available on the instrument to allow for optical/X-ray pump–probe schemes (Grychtol *et al.*, 2021 ▸; Rivas *et al.*, 2022 ▸) as illustrated in Fig. 2 ▸. The imaging option will be increasingly valuable with improved control and characterization of the XFEL pulses, especially when combined with pulse durations shorter than typical core–hole lifetimes (Serkez *et al.*, 2018 ▸).

## 2. Spectrometer design

### 2.1. Inception

In order to image the XFEL beam progression through the target while monitoring the spectral content of the emitted/scattered radiation, the optical system has two separate functions, one in the horizontal plane for 1D imaging and one in the vertical plane for energy dispersion. In this way a two-dimensional image can be recorded by the detector, containing spatial information in one dimension and spectral content in the other.

### 2.2. Imaging optics

The spectrometer operates at grazing incidence, which means that the Abbe sine condition for imaging is violated (Abbe, 1881 ▸). Therefore, Wolter optics (Wolter, 1952 ▸) is used to achieve high spatial resolution for the 1D imaging in the horizontal plane. The combination of a plane-hyperbolic mirror and a plane-elliptic mirror essentially constitutes a 1D microscope and provides good imaging properties. Wolter mirror configurations are successfully used in satellite-borne telescopes for X-ray astronomy (Champey *et al.*, 2022 ▸; Giacconi, 2008 ▸, 2003 ▸)

**Figure 3:**
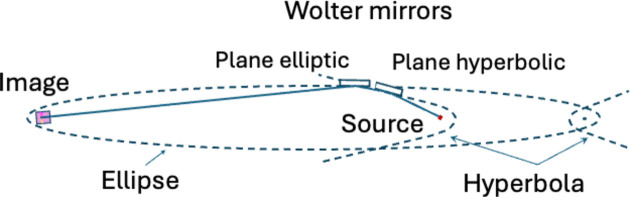
The principle of 1D Wolter optics imaging in microscopy mode. The hyperbolic mirror makes a virtual image of the source, which becomes a real object for the imaging of the elliptic mirror which gives the image on the detector. A grating for wavelength dispersion intercepts the beam path in the vertical plane, as shown in Fig. 1. See the text for further description.

The hyperbolic elliptic Wolter pair, working at 2.5° grazing angle of incidence, collects and focuses the radiation from the spatially extended source to form a magnified image on the detector. The source is placed at the nearest focus of the hyperbolic mirror, while its other focus coincides with one focus of the elliptic mirror. The detector is placed at the second focus of the elliptic mirror, as shown in Fig. 3 ▸.

The Wolter mirror parameters were chosen to yield a ten­fold magnification, given minimum distances of the source to the first mirror and of the first to the second mirror of 200 mm. This implies that a 2 mm long source will give a 20 mm long image on the detector, and that the total optical path length of the instrument is fixed to 3.8 m. The parameters of the Wolter mirrors are presented in Table 1 ▸.

### 2.3. Diffraction optics

The energy dispersion in the vertical plane is based on the Rowland geometry, using cylindrical gratings with constant line spacing. This choice offers flexibility, a broad wavelength range and simple operation in combination with 1D imaging in the horizontal plane. Use of the Rowland geometry is also motivated by the choice to use a detector based on microchannel plates (MCPs), which in turn is motivated by the time structure of the incident XFEL radiation. The EuXFEL delivers trains of photon pulses with a minimum separation of 220 ns. The stochastic SASE process implies that each pulse has different characteristics, and to allow for sorting data on pulse characteristics the time resolution of the detection must be better than the pulse separation. This is conveniently accomplished using MCP-based detectors. Although the spatial resolution in such detectors is inferior to modern CCD detectors, it is not an essential drawback. In grazing incidence, where the MCP sensitivity is much higher than CCD-based detectors, the projection of the beam cross section significantly improves the dispersion so that good energy resolution is also achieved when the spatial detector resolution is limited. This feature is exploited in the classical Rowland geometry.

Soft X-ray Rowland spectrometers are usually operated with a fixed grating and a movable detector (Nordgren *et al.*, 1989 ▸). However, the fixed focal length of the Wolter optics requires a design where the dispersive part of the instrument also operates in a fixed path-length mode. In the Rowland mount the source, grating and detector are placed on a circle with half the radius of the grating. By moving the grating, instead of the detector, along the Rowland circle, it is possible to select the energy range of the photons hitting the detector. This means that the vertex angle at the grating stays constant for all positions of the grating, owing to the edge angle theorem. The sum of the incidence and diffraction angles is 173.67° in this design, and the path length from source to detector via the grating is close to constant during the grating movement. The resulting small deviation is compensated by a small movement of the detector.

**Figure 4:**
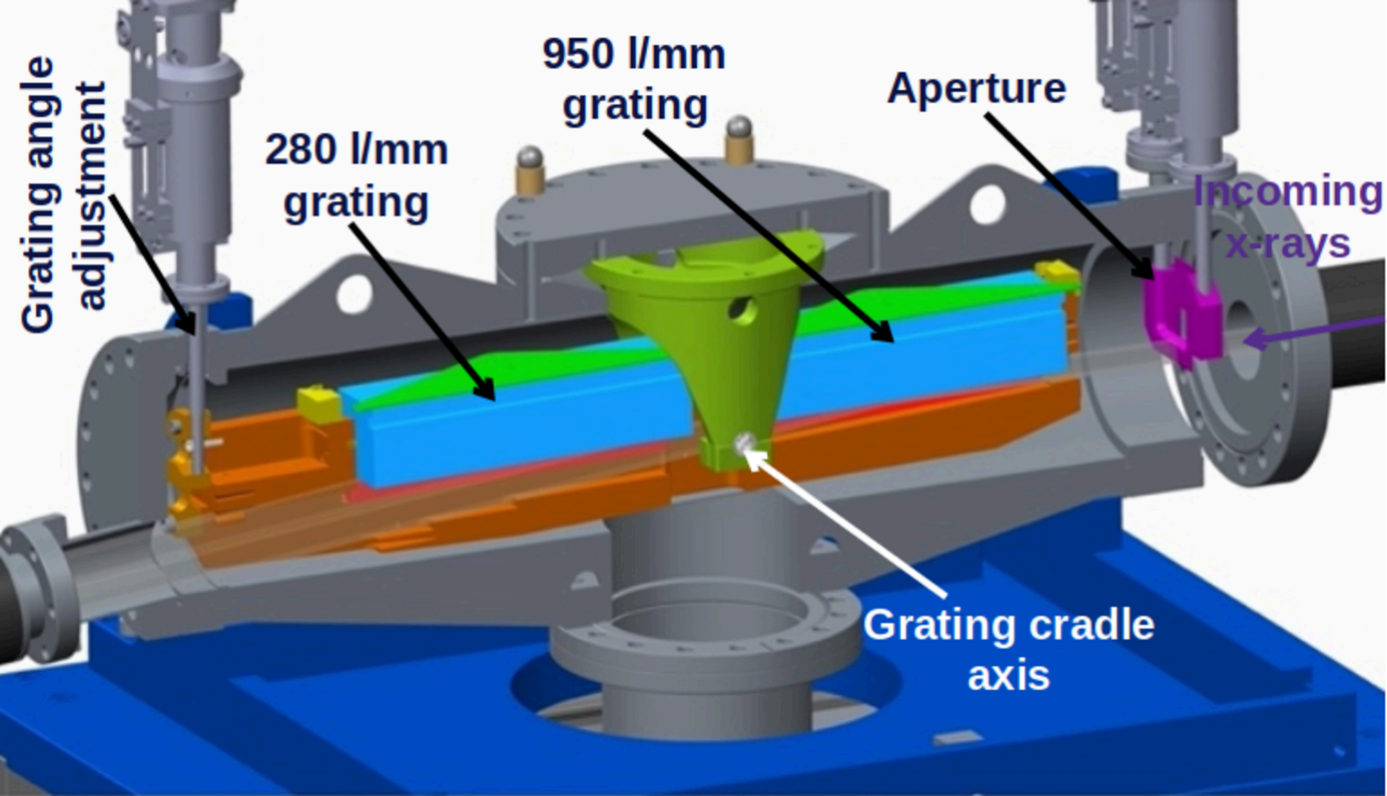
A cut view of the grating cradle with the 280 lines mm^−1^ and 950 lines mm^−1^ gratings, showing the adjustable aperture for selecting the grating cradle angle and illuminated area.

The spectrometer is equipped with two in-line mounted cylindrical gratings, one with 280 lines mm^−1^ and one with 950 lines mm^−1^. A baffle can be moved to illuminate the grating of choice, which then intercepts the beam path between the Wolter mirror pair and the detector (Fig. 4 ▸). Exploiting both first and second orders of diffraction, the arrangement covers the energy range from 145 eV up to 1400 eV with reasonable transmission and a theoretical resolving power of ∼10^4^ (see Section 4 for details). By moving slightly off the optimum positions the energy range can also be somewhat extended towards lower energies, at the cost of energy resolution.

When the grating moves away from the source the grazing-incidence angle increases, while the corresponding angle of diffraction decreases. For positive orders of diffraction this motion implies moving the energy range captured by the detector towards higher energies. This is seemingly in conflict with the notion that smaller grazing angles of incidence are wanted for higher energies, where reflectivity and thereby grating efficiency are reduced. However, going to higher positive orders of diffraction implies a smaller grazing angle of incidence, and thus the energy range can be extended to higher energies while exploiting the advantage of improved efficiency at small incidence angles. In the lower energy range, the larger grazing angles in the first positive order of diffraction do not imply significant loss of efficiency. Positive order operation was chosen in order to optimize overall performance. In principle, the spectrometer could also be operated in negative orders of diffraction, where higher energies would imply smaller grazing-incidence angles.

The grating radius (the same for both gratings) at 35 m means that the deviation between the curved focal plane (17.5 m radius) and the flat detector is small enough over the length of the detector (80 mm) not to cause broadening. Also, the tenfold magnification of the Wolter pair makes the converging angle at the detector small, and thus a sizeable depth of field is achieved.

### 2.4. Mechanical design and operation

**Figure 5:**
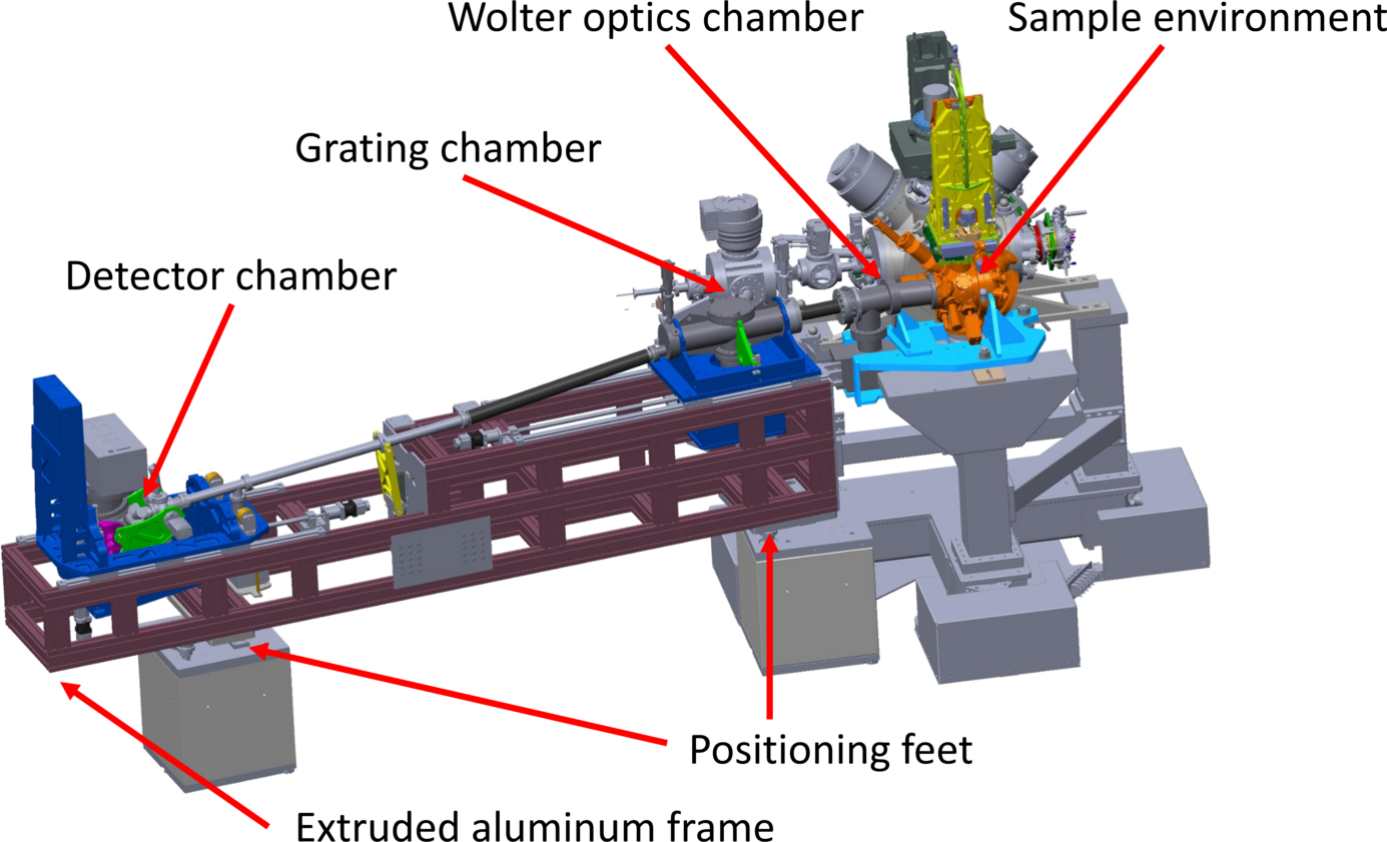
A graphical overview of the 1D imaging soft X-ray spectrometer mounted on the AQS experimental chamber of the SQS instrument. Welded bellows are mounted between the Wolter chamber and the grating chamber, and between the grating chamber and the detector chamber, and there is also a bellows internally in the detector chamber allowing for the rotation of the detector.

The spectrometer attaches to the atomic-like quantum systems (AQS) experimental chamber of the SQS instrument at the F1′ focus position (Mazza *et al.*, 2023 ▸; https://www.xfel.eu/facility/instruments/sqs/index_eng.html). The setup has three main chambers: one houses the Wolter mirror pair, a second houses the gratings and the third chamber houses the detector. The last two chambers are both movable to allow for positioning of the grating and detector on the Rowland circle. Fig. 5 ▸ shows a 3D drawing of the spectrometer.

The frame of the spectrometer is made from extruded aluminium profiles and rests on three feet on two granite blocks. The feet can be adjusted in three dimensions and, together with a spherical seat on top of each foot, this allows full six-axis alignment of the frame to the XFEL beam. During operation the detector and grating chambers are housed in the frame, while the Wolter optics is mounted fixed to the experimental chamber. In the off-line configuration the Wolter optics chamber is mounted to the spectrometer frame with a removable sub-frame.

### 2.5. Wolter optics chamber

The two Wolter mirrors are mounted fixed to a monolithic aluminium structure using three stainless steel balls for each mirror, touching the optical surface outside the active area. The relative alignment of the mirrors is defined by the machining precision. Similarly, the transverse and longitudinal positions of the mirrors are defined by pairs of stainless steel balls, spring-loaded from one side in the monolithic aluminium structure. A spring-loaded back plate makes sure that the mirror is in contact with the front steel balls.

**Figure 6:**
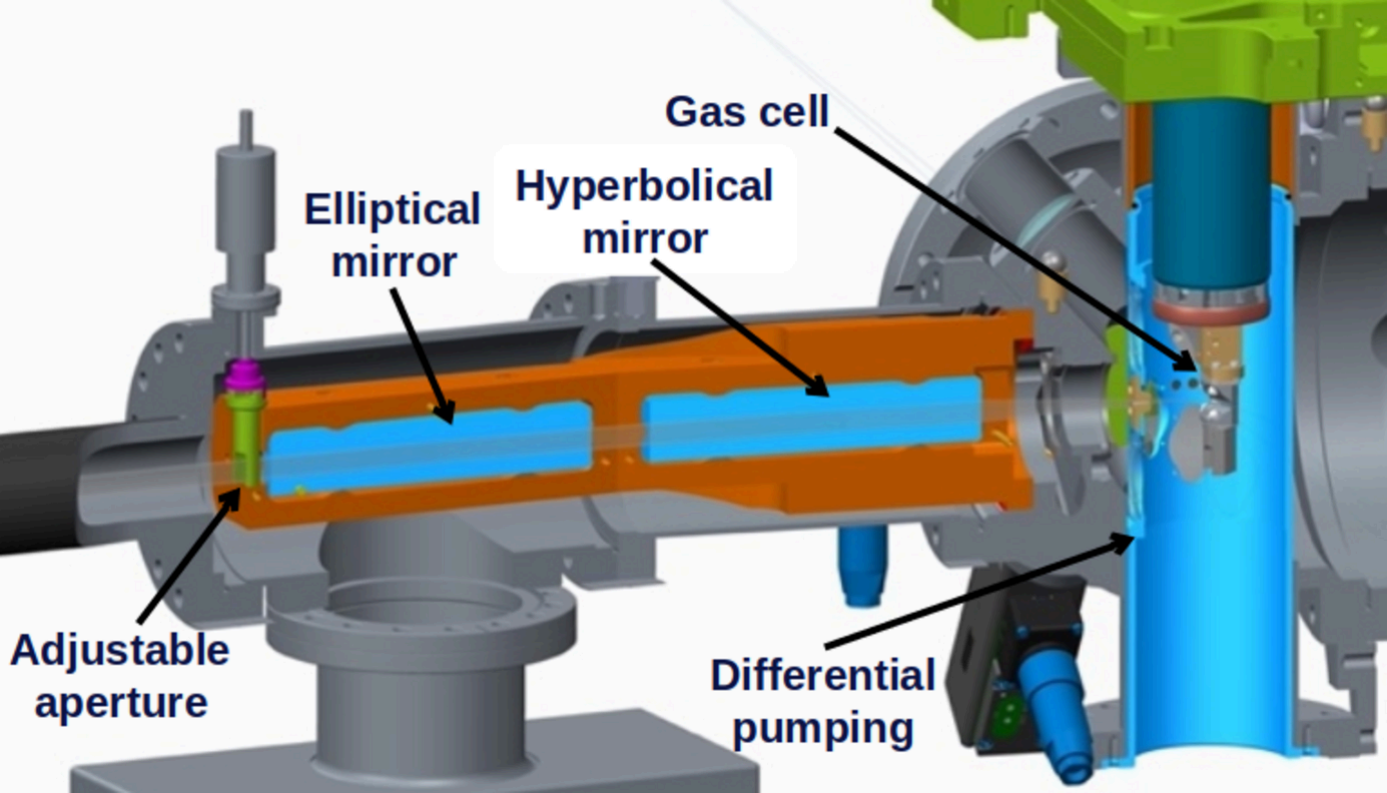
A cut view of gas cell arrangement and Wolter optics mounting. The acceptance angle is adjustable by rotation of a fork baffle. In the right-hand part of the figure, the sample gas cell with the differential pumping stage is shown.

Since the Wolter optics entrance arm is short (200 mm) and the space around the experimental station is limited, the monolithic holder of the Wolter optics is mounted directly on a reference surface machined into the receiving CF flange on the experimental chamber. This in turn sits on a six-axis motion table (Newport, Irvine, California, USA; https://www.newport.com/s/assemblies-xy-stages-xyz-stages-gimbals) together with the AQS experimental chamber of the SQS instrument, dispensing with the need for additional alignment kinematics for the Wolter optics. Downstream of the Wolter optics is an adjustable aperture controlling the horizontal acceptance angle. The enclosing Wolter mirror chamber is mounted fixed to the experimental chamber (Fig. 6 ▸) and is pumped by a turbo molecular pump. The gas load in the spectrometer can be reduced by introducing a thin membrane mounted in a VAT mini ultra-high vacuum valve, situated in between the experimental chamber and the Wolter chamber in a custom mount.

### 2.6. Grating chamber

**Figure 7:**
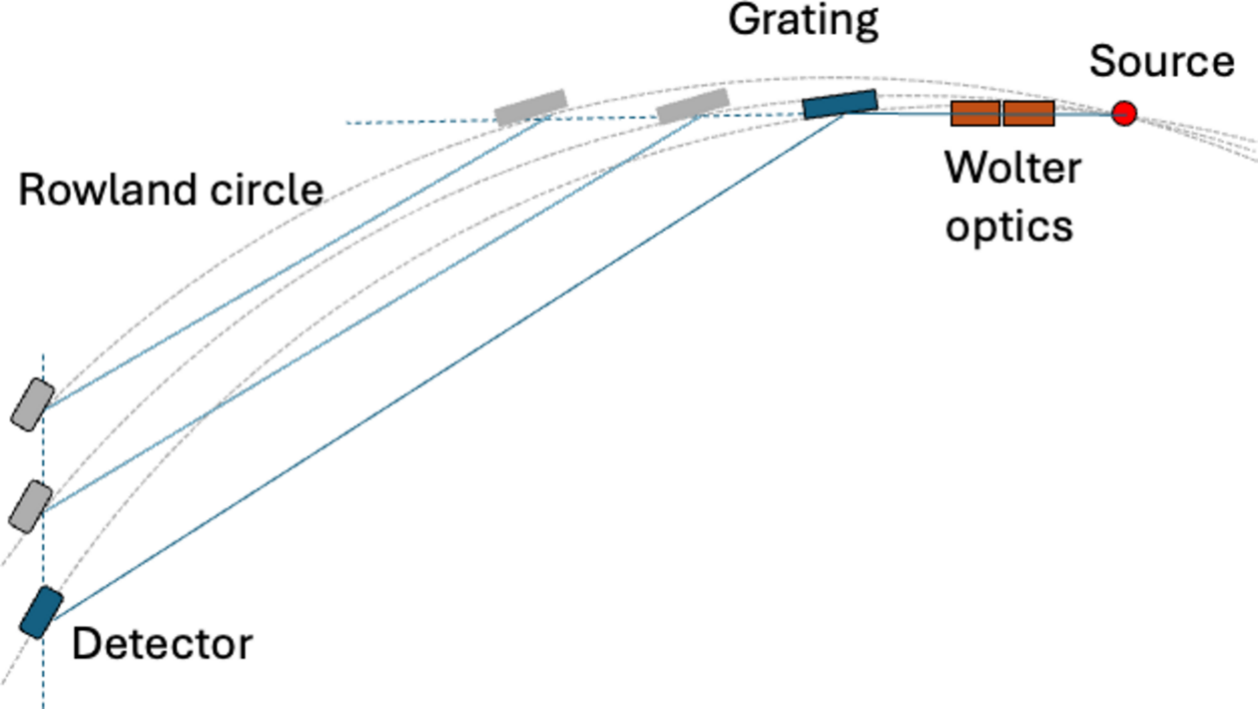
The geometry of the spectrometer in the vertical (dispersive) plane, showing the principle of operation for choosing the photon energy range (not to scale, for clarity). Setting a particular incidence angle is done by horizontal movement and rotation of the grating cradle. Since the horizontal grating movement rotates the Rowland circle around the source, the detector needs to be correspondingly shifted vertically and rotated to maintain its tangential orientation to the Rowland circle for a given grating cradle setting.

The energy range is chosen by moving the grating on the Rowland circle, *i.e.* simultaneously varying the position and incidence angle, while keeping the source fixed and changing the detector position only a little to comply with the Rowland criterion. This keeps the path length essentially constant, as required by the fixed focal length of the Wolter optics. As the focusing properties are sensitive to the position and orientation of the grating, this calls for simple and stable motion control. Therefore, the motion is executed as a linear horizontal motion of the entire grating chamber and an angle adjustment of the mirror holder inside the chamber, dispensing with the need for a sensitive vertical shift mechanism. This effectively causes the Rowland circle to rotate around the source instead of staying fixed in space. The light emitted from the target is essentially collected horizontally for all grating positions. The rotation of the Rowland circle requires a small corresponding detector translation, as well as a rotation to align with the Rowland circle, as shown in Fig. 7 ▸.

The two gratings are, like the Wolter mirrors, mounted on a monolithic aluminium cradle (Fig. 4 ▸) using precision machined reference surfaces and steel balls. This defines the optical surfaces of the gratings in relation to the cradle, which is suspended inside the vacuum chamber by two flexure bearings (Riverhawk, New Hartford, New York, USA; https://www.flexpivots.com/about-us/) allowing rotational adjustment controlled by a sine-arm arrangement. The angular motion is monitored by a linear scale that is bent to a cylindrical shape centered on the point of rotation, allowing it to be read as a tangential motion by a Renishaw linear absolute encoder (Renishaw, Wotton-under-Edge, Gloucestershire, UK; https://www.renishaw.com/en/position-encoders--6331).

The grating chamber with its attached ion pump runs on precision rails on top of the spectrometer frame (SKF, Gothenburg, Sweden). The horizontal motion of the grating chamber is actuated by a linear translator controlled by a stepping motor with a linear absolute encoder (Renishaw). The chamber mount allows for roll adjustment of the gratings relative to the frame. The roll is controlled by a fine-threaded screw and a lever arm. A digital level meter is used to align the roll between the gratings and the detector via an external reference surface. The axis of rotation of the grating cradle is at the mid-point between the two gratings. This means that a small vertical translation of the grating has to be accounted for when calculating the angle of incidence setting. Grating selection is made by two independently movable blades in front of the grating assembly. This aperture is used for grating selection as well as controlling the length of illumination in order to set a desired match between transmission and resolution (see Section 4). The blades are operated by motorized linear feedthroughs and monitored by external absolute encoders (Renishaw).

### 2.7. Detector chamber

The detector chamber is mounted on a similar carriage to the gratings, resting on rails mounted to the frame, allowing horizontal motion for alignment. Inside the carriage a vertical translation table is mounted, holding the detector chamber, and allowing vertical movement. The detector chamber is mounted with two flexure bearings (Riverhawk), also allowing rotational adjustment around a horizontal axis through the detector surface perpendicular to the incident light. The MCP detector is mounted on a CF flange on top of the detector housing so that it can be easily removed without interfering with the positioning system. All motions of the detector are monitored by absolute encoders (Renishaw). In front of the detector housing a small CF 35 chamber houses a filter manipulator, enabling attenuation of the signal and a reduction in the count rate, effectively increasing the dynamic range of the detector. A reduction in the count rate can also be accomplished by closing the apertures that limit the illumination of the optics.

### 2.8. Vacuum system

The spectrometer is pumped by three vacuum pumps: a turbo molecular pump (300 l s^−1^) at the Wolter chamber, an ion pump (75 l s^−1^) at the grating chamber and an ion pump (40 l s^−1^) at the detector chamber. The grating chamber is connected to the Wolter chamber and the detector chamber by means of edge-welded bellows to allow 700 mm scanning length. A beam pipe is used for part of the distance to the detector chamber as this distance is considerably longer than the distance between the grating chamber and the Wolter chamber. As the detector moves in height, this pipe is adjusted in height by a vertical motion table. The ion pump in front of the detector chamber is adjusted vertically by a spring-loaded platform, which reduces its load on the system.

The experimental chamber is designed as part of the spectrometer, as it is directly connected to the Wolter mirrors. There are two valves between the Wolter mirrors and the interaction region: one vacuum valve to separate the spectrometer from the experimental chamber during venting, and one valve containing a thin membrane to separate the vacuum in the spectrometer from the vacuum in the experimental chamber during operation. The experimental chamber is mounted as an in-line intersection on the SQS instrument. It houses a Roots pump-assisted differential pumping stage, which in turn houses a sample gas cell. Small orifices in the differential pumping stage allow the XFEL beam and the scattered radiation to pass. These orifices can be exchanged to allow different sizes depending on the beam size and pressure. These orifices can also be made to allow in-coupling of the nearly collinear laser beam. The main experimental chamber is pumped by a 1200 l s^−1^ turbo molecular pump mounted on a CF 200 flange.

### 2.9. Sample environment

**Figure 8:**
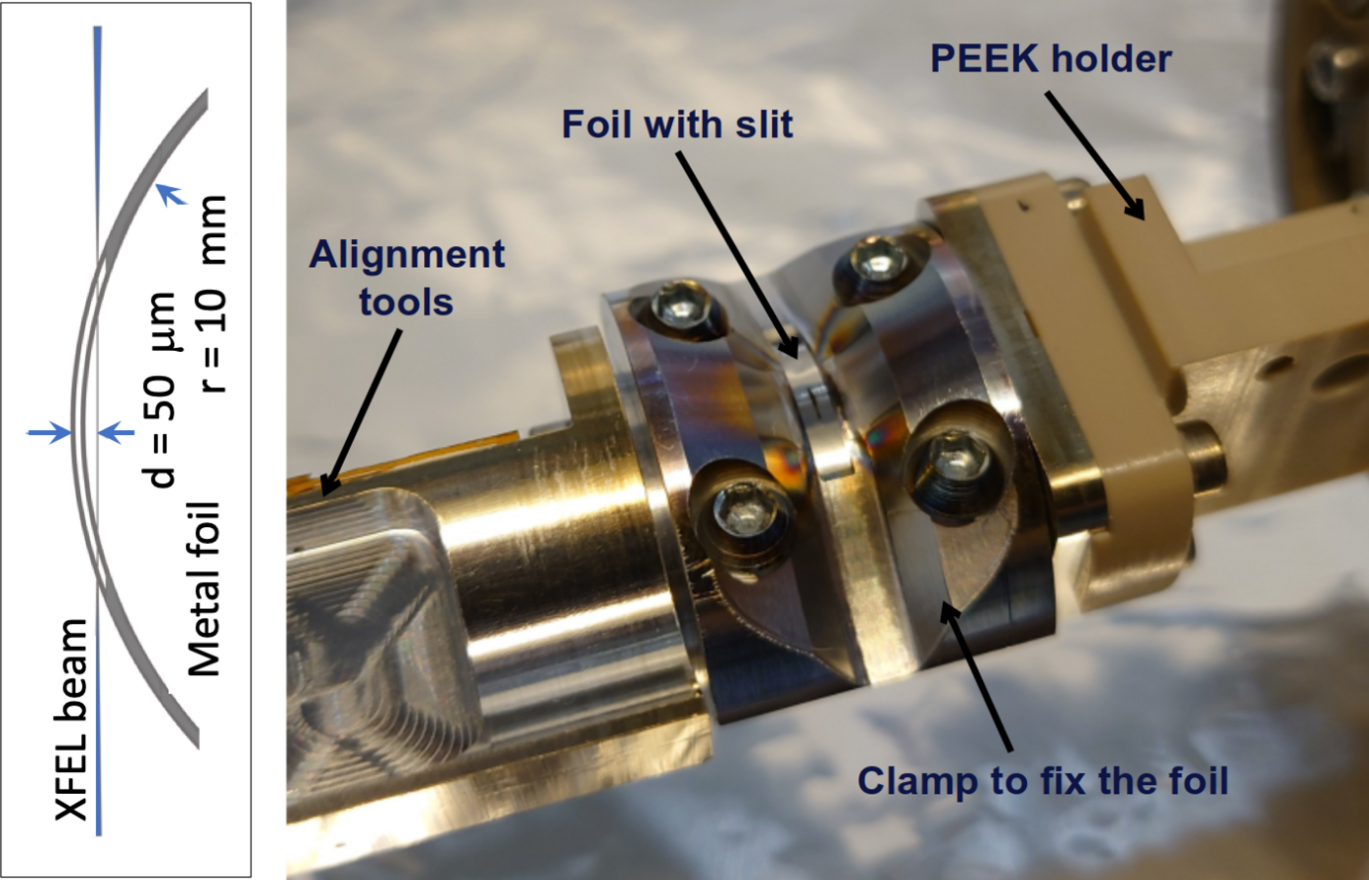
A picture of the gas cell arrangement. A thin metal foil enclosing the gas compartment is cut by the XFEL beam to produce a beam-sized slit. The length of the slit is defined by the lateral movement of the foil during etching (nominally 2 mm long).

The experiment requires a gas target with high pressure and a steep pressure gradient so that the interaction region is well defined in terms of absorption/interaction length. In addition, it is desirable to avoid tedious alignment procedures. For this purpose a gas cell has been designed that features a thin curved metal foil, in which the XFEL beam cuts a slit at the start of the experiment (Fig. 8 ▸). In this way, the sample gas becomes available for the XFEL beam in a well defined gas sheet close to the slit (∼50 µm), as seen from optical fluorescence monitored with a microscope. As the slit appears where the X-ray beam is present, no further alignment of the cell is needed. The cut slit is sufficiently small to accomplish measurements at high gas pressure in the gas cell while still maintaining an operational vacuum though differential pumping.

The cutting procedure is facilitated using a high-precision four-axis manipulator (Agåker *et al.*, 2021 ▸) attached on top of the experimental chamber. The cell body is made of aluminium and the gas compartment is enclosed by a metal foil, bent to a cylindrical shape of 10 mm radius and sealed to the main body of the gas cell (Fig. 8 ▸). Efficient pumping by means of the differential stages makes sample pressures up to 1 bar feasible, while maintaining sufficient vacuum conditions in the beamline and in the spectrometer. During the cutting an upstream membrane is introduced to protect the refocusing mirrors of the beamline.

The gas cell arrangement is also made to allow the near-collinear laser beam to enter the interaction region at a 15° angle to the XFEL beam in order to facilitate pump–probe experiments in the imaged section without the need for an external delay stage (Fig. 2 ▸). Since the laser beam projection will propagate slightly slower in the direction of the XFEL beam due to the angle, there will be a stretching of the time scale. A 15° angle corresponds to 1.8 fs between each 15 µm segment along the beam, assuming both beams propagate at the vacuum speed of light, and a time window of 0.24 ps for the 2 mm imaged path length. This will be affected by a difference in refractive indices for the two beams, a difference that could also be due to nonlinear interaction in the medium.

## 3. Detector system

The optical system of the spectrometer produces a 1D image of the XFEL beam path in one direction, and photon energy dispersion in the perpendicular direction. This calls for a 2D position-sensitive detector. Furthermore, the time structure of the XFEL puts unique demands on the detector system since the X-ray pulses come in bursts of up to 2700 pulses at 10 Hz with a minimum separation between pulses of 220 ns (Decking *et al.*, 2020 ▸). The pulse duration is typically about 25 fs, and due to the nature of the SASE process the pulses are not identical and single pulse spectra need to be saved separately to allow for detailed analysis. The arrival time of the emitted photons is essentially dictated by the photon path length from the sample to various positions on the detector surface, so that all X-rays from a single XFEL pulse hit the detector within a few hundred picoseconds.

**Figure 9:**
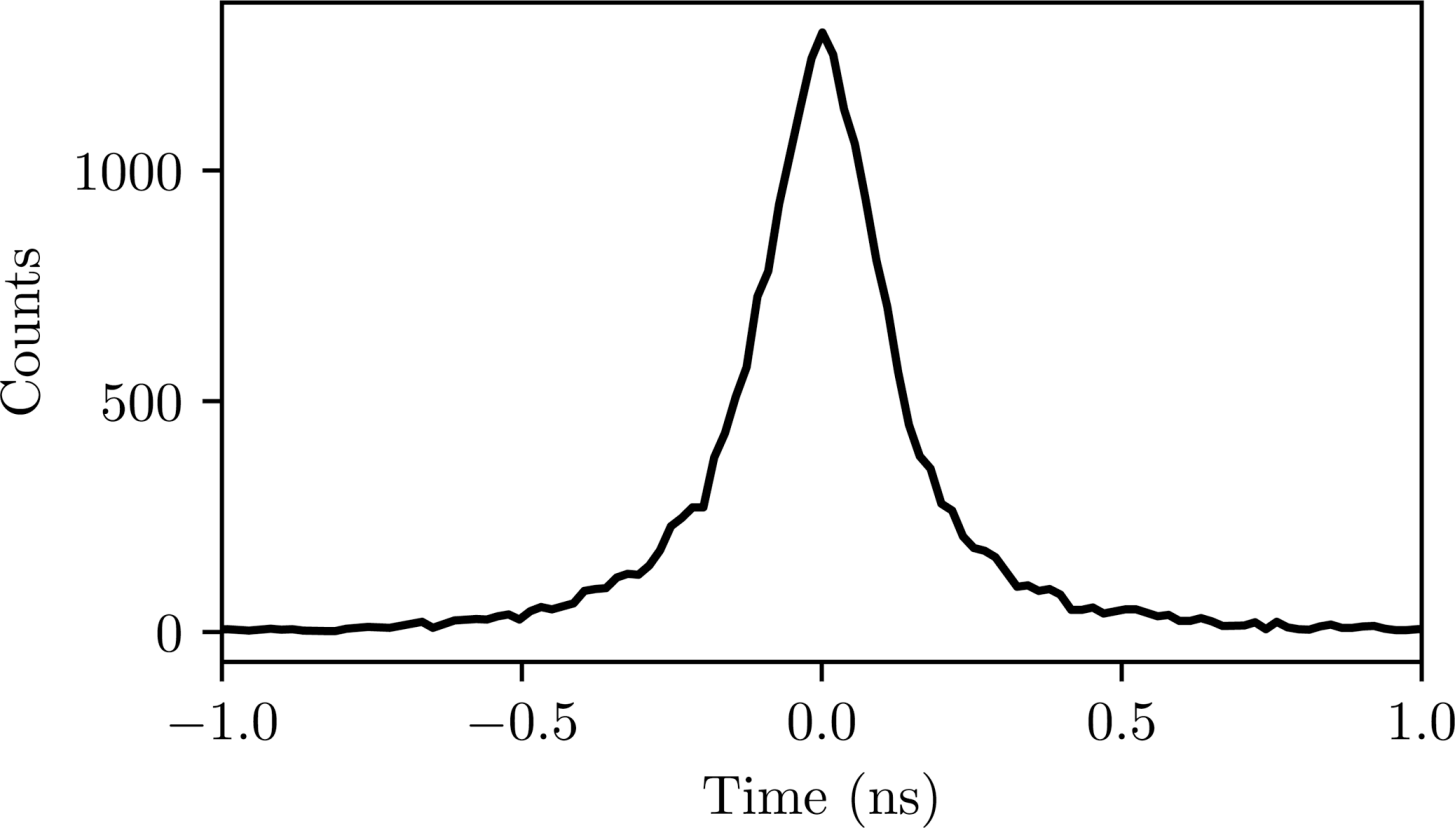
The time distribution of the detected photons during an experiment on the SQS instrument. The intrinsic time resolution of delay-line detectors allows reduction of dark noise as well as studying the time evolution of scattering processes on the corresponding time scale.

The minimum separation of the XFEL pulses, 220 ns, therefore sets the requirement for readout time. To comply with this demand we have chosen a 2D resolving MCP-based delay-line detector (Surface Concept, Mainz, Germany; https://www.surface-concept.com/) using two crossed delay lines. This limits the count rate to below 1 count per pulse, which still gives reasonable count rates due to the high repetition rate of the EuXFEL. The delay-line detector offers a means of obtaining timing information at the level of about 200 ps (Fig. 9 ▸). This is an important feature as it allows background reduction, and it can also be used to investigate scattering dynamics on this time scale. Improved detection capabilities are considered for future upgrades.

## 4. Ray tracing

The optical performance of the spectrometer was studied by means of ray-tracing simulations at different configurations and photon energies using the *RAY UI* ray-tracing software (Baumgärtel *et al.*, 2016 ▸).

Source files were generated to simulate an extended source with varying properties along its length by the use of multiple source segments, with various properties in terms of size, spatial distribution and energy content. Eleven individual sources spread over 2 mm in 0.2 mm steps, at positions −1, −0.8,…0,…0.8, 1 mm along the XFEL beam were used in the simulations. Each source has a 1 µm × 1 µm × 15 µm (W × H × L) FWHM Gaussian spatial distribution, and emits rays homogeneously in a 26 mrad horizontal and 4.6 mrad vertical wedge towards the spectrometer. This solid angle is chosen to match the full illumination of the optics, which corresponds to a total acceptance of ∼4π × 10^−5^ sterad. Each source has a central energy that is varied over the energy range of the instrument, *i.e.* for photon energies between 145 eV and 1400 eV, and two side bands separated by ±0.5 eV from the central energy. Additionally, each source energy has a Gaussian FWHM energy spread of 20 meV. In the simulations, surface errors for all optical elements are used according to the specified values.

Output from *RAY UI* in the form of rays reaching the detector element, *i.e.* a 2D image of the ray distribution, was analyzed in a similar way to how real counts on the detector would be. The detector bin sizes were set to 15 µm × 30 µm [imaging (*x*) × dispersive (*y*) direction].

**Figure 10:**
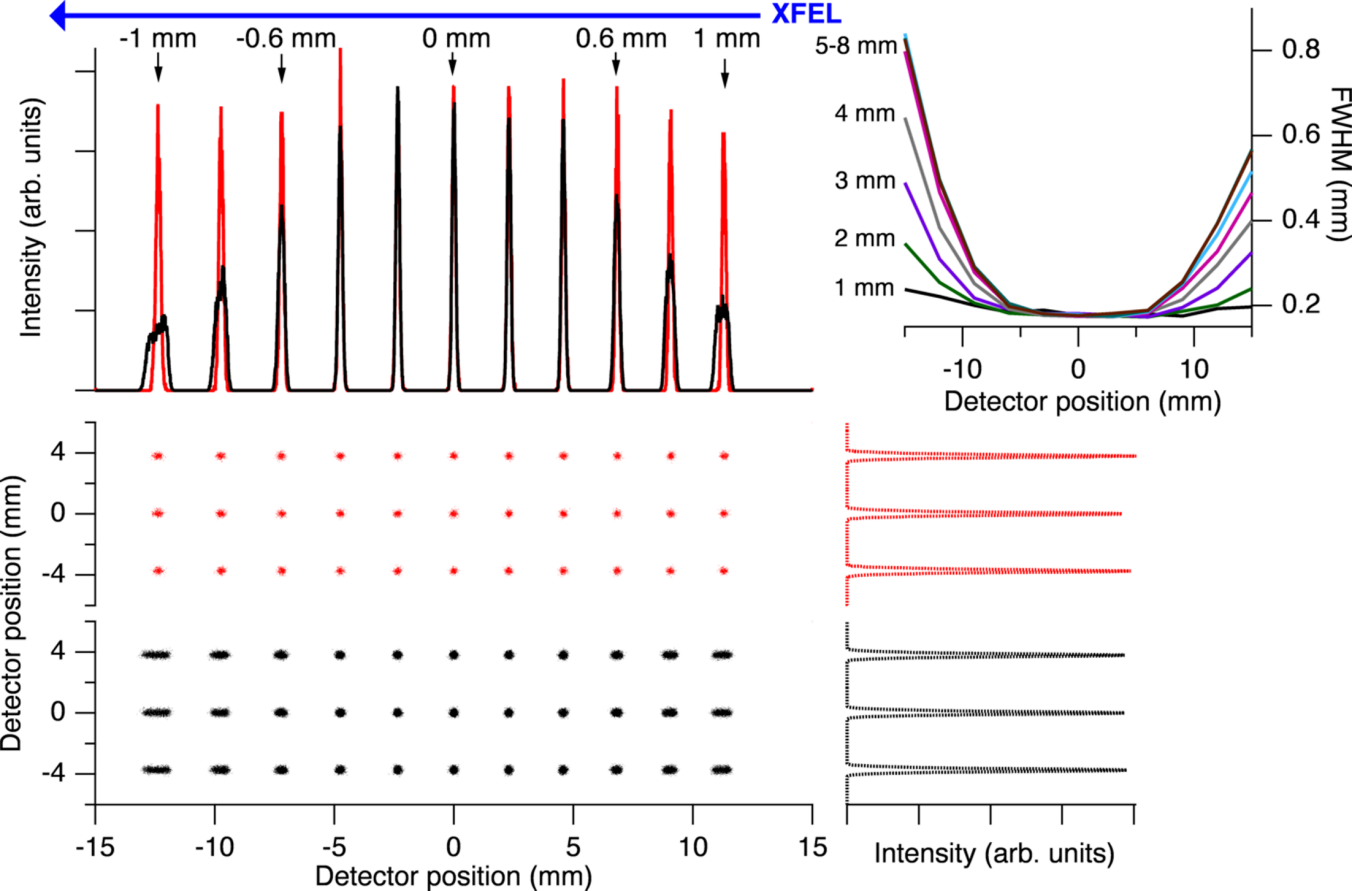
The middle and bottom left-hand panels show the detector images of a source with 11 source sections, emitting photon energies at 499.5, 500.0 and 500.5 eV. In the central panel, the aperture defining the illumination of the Wolter mirrors is set to 1 mm, corresponding to 1/6 of full illumination (color code: red). The bottom panel shows the results for full illumination (color code: black). In the top left panel, projections of the two detector images are shown in the imaging direction, and projections in the dispersive direction are shown in the middle and bottom right-hand panels. In the top right panel, the widths of the 11 peaks in the imaging direction are shown for openings 1–8 mm of the Wolter mirror aperture. The decrease in transmission when the Wolter optics aperture is closing is directly reflected in the two detector images. All projected images are normalized to the same total intensity, facilitating comparison of resolution. The energy resolution (middle and bottom right-hand panels) is not affected by the Wolter optics aperture.

In the lower and middle left-hand panels in Fig. 10 ▸, two detector images are shown resulting from rays emitted from the 11 sources along the beam direction. In the horizontal (*x*) direction this is a magnified image of the sources and in the vertical (*y*) direction the energy dispersion is shown.

The two 2D images show results using different apertures for the Wolter optics (bottom panel: fully open, and middle panel: closed to a 1 mm horizontal aperture). The central part around 0 mm on the horizontal scale is the same in the two cases but at the edges the lower panel (full illumination of the Wolter optics) has much wider dots in the horizontal direction due to aberrations in the spectrometer. By reducing the aperture after the Wolter optics the spatial resolution at the edges can be improved, at the cost of transmission. At the top left of Fig. 10 ▸ the projections of the sources are shown for both illumination cases (black: full illumination, red: 1 mm aperture, corresponding to 1/6 of full illumination). The lower right-hand panels show the projections of the 2D images in the energy dispersion direction, confirming that the aperture setting for the Wolter optics does not affect the energy resolution.

In the top right-hand corner of Fig. 10 ▸ the widths of the source images in the spatial direction are shown as a function of the Wolter optics aperture (1, 2, 3, 4, 5–8 mm). It is mostly the three outermost peaks on each side that are affected by the aperture opening, corresponding to the outermost 0.5 mm on each side, while the central peaks are mostly the same, corresponding to the central 1 mm of the source. For the real conditions of an experiment a compromise between overall intensity in the spectral analysis and spatial resolution in the imaging direction has to be found.

**Figure 11:**
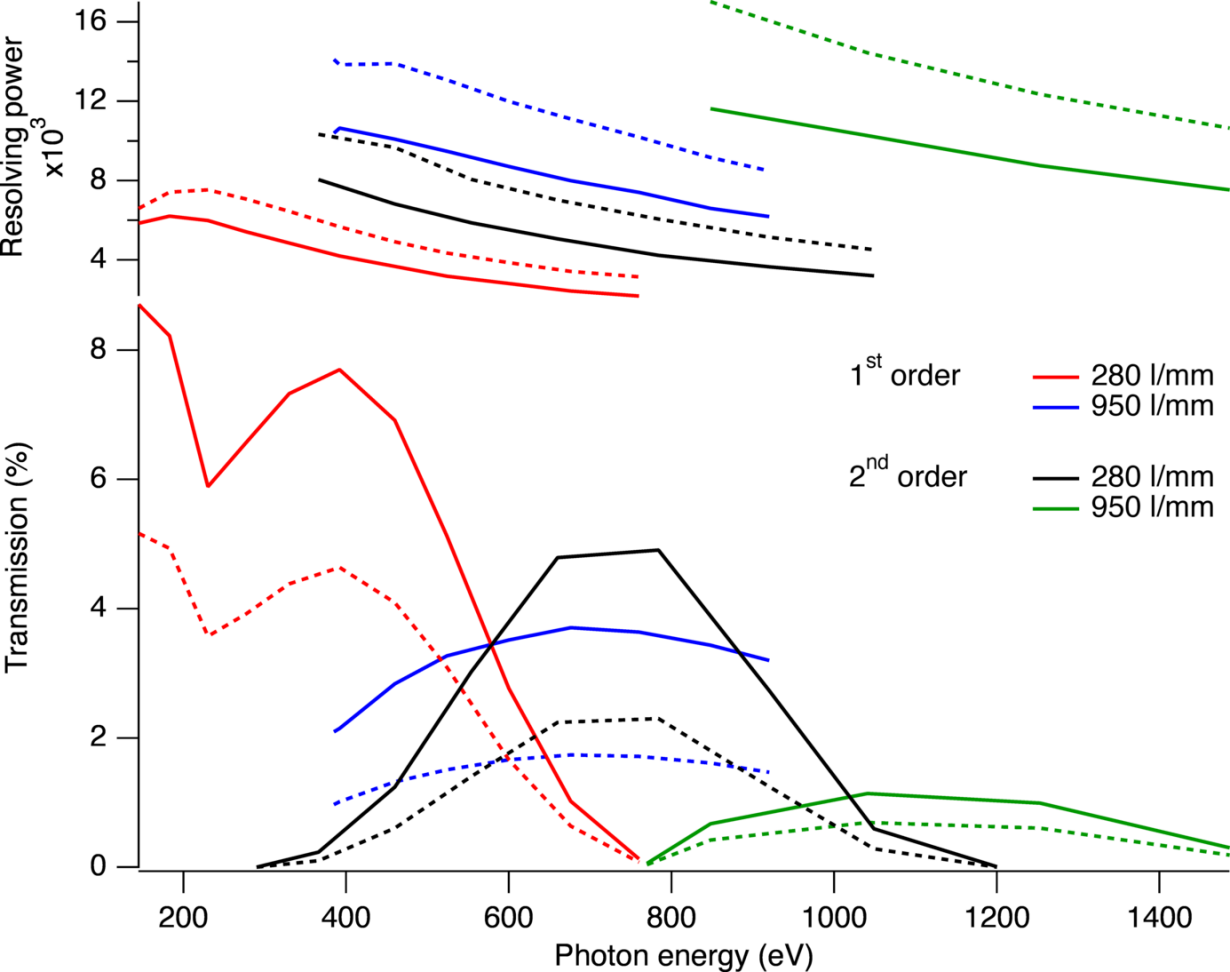
The resolving power (top panel) and the transmission (bottom panel) using the two gratings (280 lines mm^−1^ and 950 lines mm^−1^) in first and second orders of diffraction as a function of photon energy. Solid lines correspond to fully open apertures while dashed lines show the results for ‘optimal illumination’, where further reduction of the illumination does not improve energy reslution. This occurs in all configurations for an illumination of around 70–90 mm on the 150 mm long gratings.

Fig. 11 ▸ shows the transmission (lower panel) and resolving power (upper panel) of the instrument for the two gratings (280 lines mm^−1^ and 950 lines mm^−1^) in first and second orders of diffraction. For full illumination (solid lines) the resolving power is limited by aberrations. By reducing the illumination to ‘optimal illumination’ (Fig. 11, dashed lines), the reduced transmission due to further closing of the aperture is not accompanied by any significant increase in resolving power. In all cases, the optimal illumination of the 150 mm long gratings (*cf.* Table 1 ▸) is in the 70–90 mm range.

The energy resolution was determined by fitting Gaussian functions to the three energy peaks for each central energy. From the positions of the two side bands and the fact that they are 1 eV apart, the dispersion relation at the specific central energy could be determined and the ratio of the central peak energy and central peak FWHM was used to determine the resolving power (top panel in Fig. 11 ▸).

Note that the second-order transmission appears relatively large compared to the transmission in first order in this instrument. This is because the second order of diffraction implies a larger angle of incidence on the grating, which improves throughput at higher energies.

At low energies (<400 eV) the 280 lines mm^−1^ grating in first order is used. At higher energies (>400 eV) it is possible to use either the 950 lines mm^−1^ grating in first order or the 280 lines mm^−1^ in second order. Above 600 eV the 280 lines mm^−1^ grating will give slightly higher transmission but with lower resolution. Above 1000 eV the 950 lines mm^−1^ grating would be the choice.

## 5. Experimental characterization

**Figure 12:**
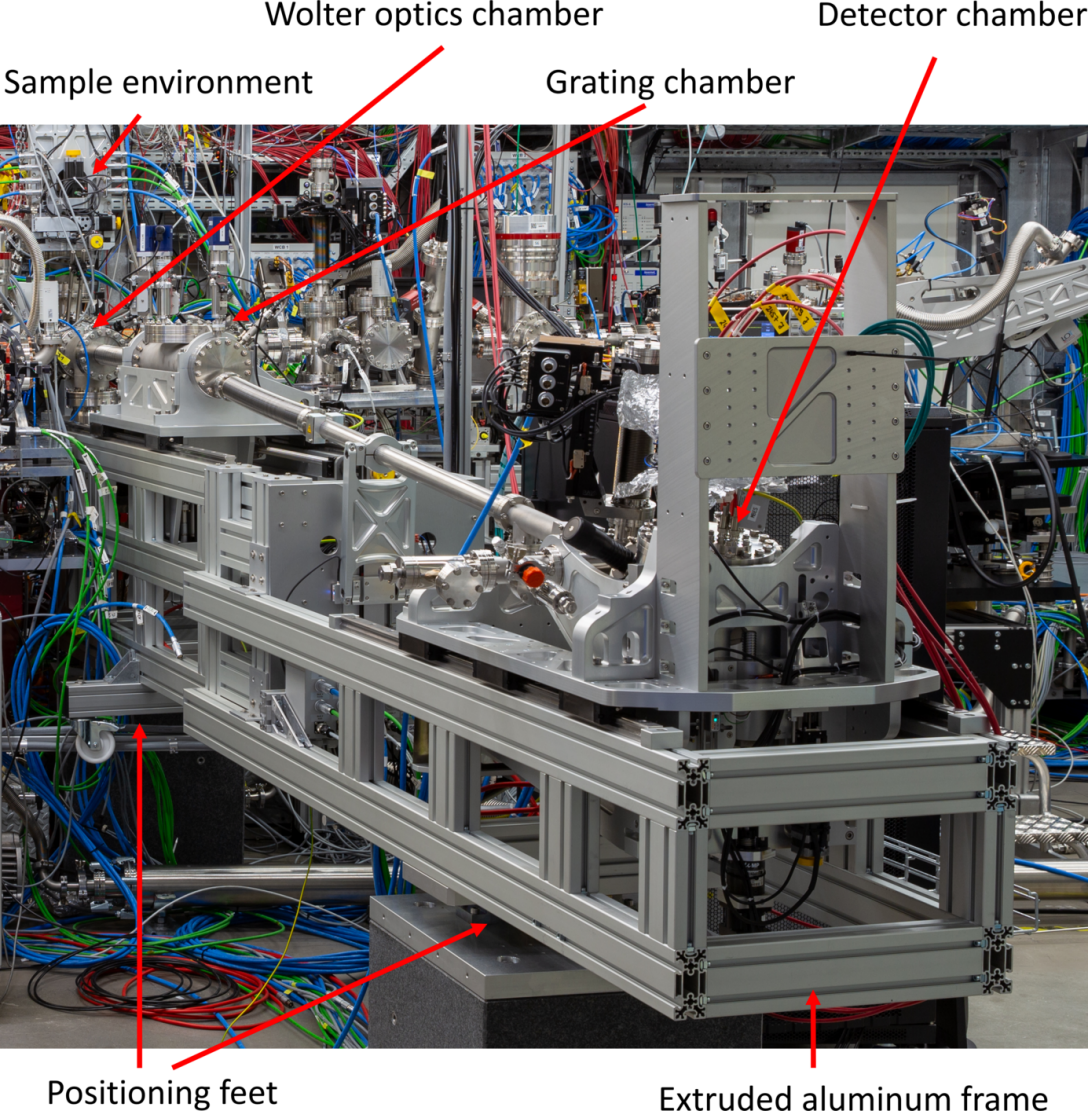
The 1D imaging soft X-ray emission spectrometer installed on the SQS instrument of the European XFEL. The detector housing is seen in the foreground (right in the picture), with the stages that allow for stable rotation and translation of the detector. The beam pipe can be moved in the vertical direction with an actuator (the middle of the picture) to follow the motion of the grating chamber (left in the picture, connected with bellows on both sides) when it slides to capture various parts of the spectrum. The Wolter optics is mounted directly in the experimental chamber, which is seen at the far back (left) below the high-precision manipulator.

The 1D imaging spectrometer was installed (Fig. 12 ▸) on the SQS instrument in 2022. During its initial commissioning, the overall operational concept was demonstrated and the performance, mainly with respect to energy resolution and imaging capabilities, was characterized. In the following some proof-of-principle results from this initial commissioning campaign are shown.

**Figure 13:**
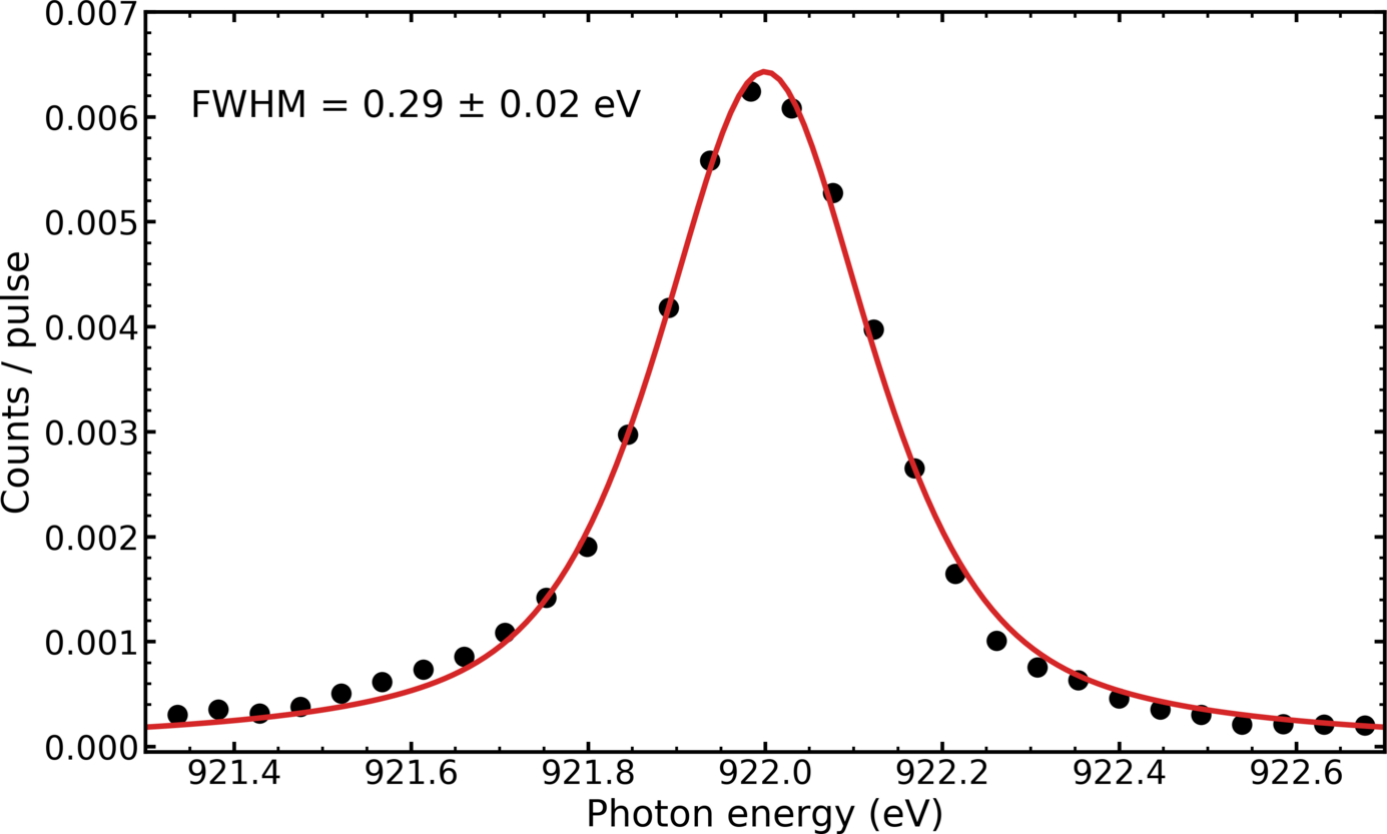
The measured *K*α emission line of Ne^8+^, showing a FWHM of 0.29 eV giving a resolving power of ∼3200. This width is larger than expected from the simulations shown in Fig. 11 where a resolving power of ∼11100 is shown. This emission line is expected to have very small intrinsic broadening, and we attribute the observed broadening as being due to *e.g.* a source size larger than the XFEL beam FWHM, or a misalignment of the optical components in the spectrometer.

In Fig. 13 ▸ an emission line assigned to *K*α transitions (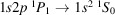) in the He-like Ne^8+^ ion is shown. The spectrum was recorded by focusing intense XFEL pulses of 4 mJ pulse energy from the SASE3 soft X-ray undulator into the gas cell at a neon pressure of 300 mbar. The EuXFEL operated in the typical 10 Hz burst mode, delivering each 400 pulses at an intra-burst repetition rate of 1.1 MHz to the SQS instrument. The photon energy was set to 1500 eV. At these high intensities the sample gas is multiply ionized and charge states up to Ne^9+^ were observed. The observation of the 2*p* → 1*s* transition in the Ne^8+^ ion, which can be well described by a Voigt line profile, demonstrates directly that fluorescence spectroscopy can be used as a tool to obtain detailed information on the excited states of the highly charged ions produced in the interaction volume, which are difficult to extract by other means, *e.g.* by electron spectroscopy. The total acquisition time of the spectrum was 80 s.

As the recorded line corresponds to a single transition and is broadened only by the finite lifetime of the upper state, the intrinsic width is expected to be very small. Thus, the measured line shape reflects the instrumental broadening. The width of the line, measured using the 950 lines mm^−1^ grating in the second order of diffraction, has a FWHM = 0.29 eV, which is larger than expected, assuming perfect alignment and an optimal source size set by the focused incident beam. With further commissioning, we aim for improvements to approach the resolving power predicted by the ray tracing (Fig. 11 ▸).

**Figure 14:**
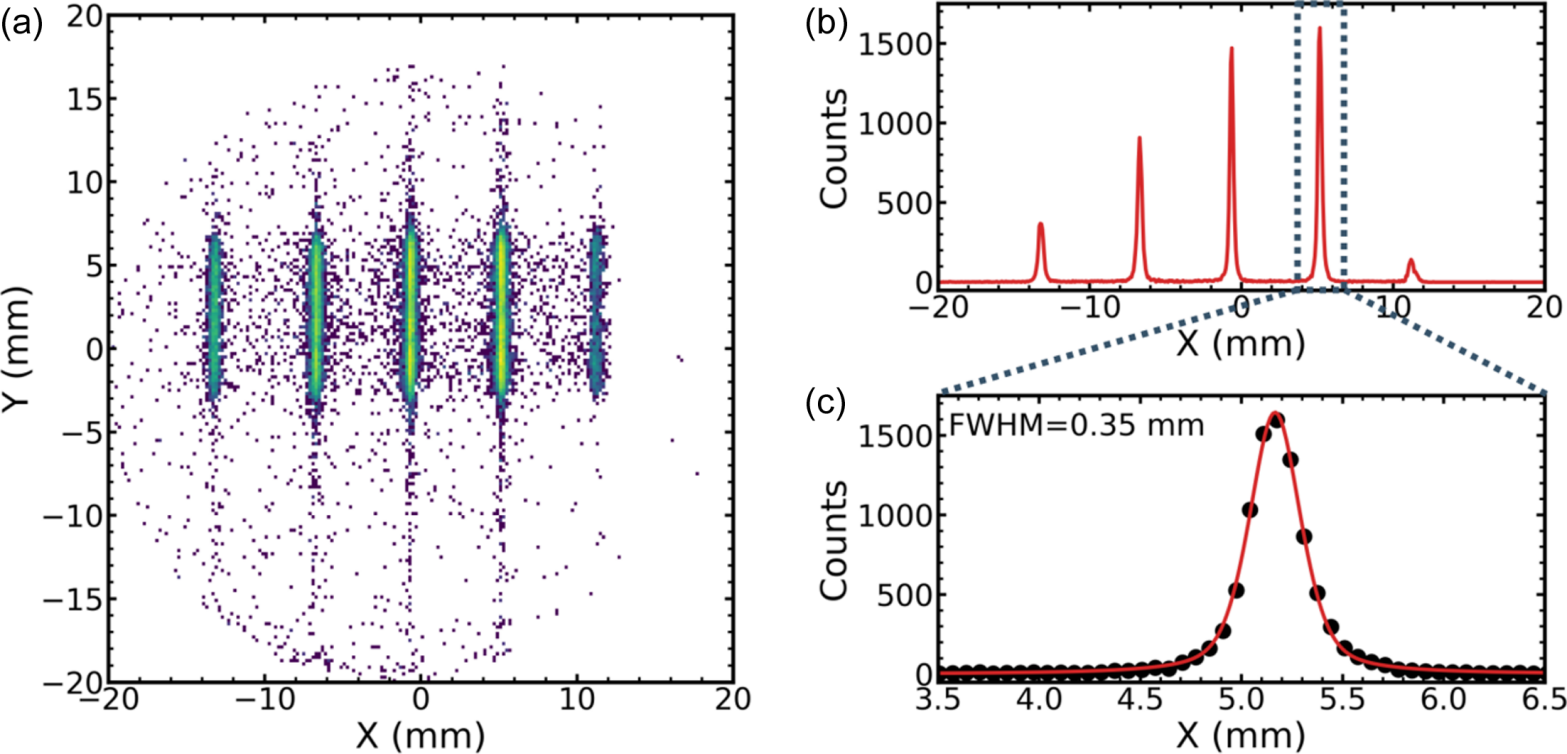
(*a*) A detector image of the Al *K*α line as the sample is moved to various positions along the XFEL beam to test the imaging capabilities of the spectrometer. The vertical energy scale is not relevant here since the beam was intentionally defocused vertically to reduce intensity, and the energy resolution is therefore limited. (*b*) Curves achieved by projecting the image on the horizontal scale. (*c*) An enlarged projected image with curve fitting. The FWHM = 0.35 mm refers to the appearance on the detector. With a tenfold magnification this corresponds to 35 µm along the beam. The non-symmetric appearance is due to the slight off-center position of the source.

For a demonstration of the imaging capabilities a solid Al target, installed at the position of the gas cell, was illuminated with a strongly attenuated XFEL beam at a photon energy of 1580 eV and the Al *K*α emission was recorded in grazing-out geometry. The corresponding detector images are shown in Fig. 14 ▸. The XFEL beam was focused horizontally and de­focused in the vertical direction to reduce intensity. By using small angles for the emitted radiation, the source is kept small in the propagation direction, and the spatial resolution is monitored as the sample moves along the beam. The detector images reflect directly in the horizontal direction the separation of the emission lines for different positions of the XFEL beam on the Al target.

Quantitative analysis of the recorded images shows that the widths of the features are slightly broader than expected from the simulation using a small source size (FWHM = 15 µm). We attribute this (again) to an extended source on the Al target and to possible remaining misalignment of the optical components. With tenfold magnification, the measured width (FWHM = 0.35 mm) is compatible with an effective source size of 35 µm. To characterize the imaging capabilities further, we plan to use a sample with well defined structure in the near future. The non-symmetric appearance of the line intensity results from a slight off-center position of the source with respect to the optics.

## 6. Conclusion

A 1D imaging soft X-ray spectrometer has been installed and commissioned on the SQS instrument of the European XFEL. The spectrometer combines high-resolution X-ray emission spectroscopy with imaging along the incident beam path through a gaseous target. The design parameters allow for detailed studies of nonlinear X-ray physics and ultrafast dynamics. This is especially powerful when combined with intense pulses of short duration, or two pulses of different energy with controlled femtosecond delay. In X-ray/laser pump–probe experiments the spatial resolution can be used to resolve small time delays.

The initial results demonstrate that the 1D imaging spectrometer is already performing at a level where unique and important experiments can be performed. It is now a part of the SQS instrument portfolio of techniques, and is available to users via the regular calls for beamtime proposals.

## Figures and Tables

**Table 1:** Parameters of the optical components and dispersive system of the 1D imaging soft X-ray spectrometer Optics material is Si〈100〉.

Shape	Size (mm)	Optical surface (mm)	Parameters (mm)
Plane-hyperbolic	170 × 30 × 40	150 × 10	Major axis −117.94, minor axis 12.879, eccentricity −319.853, sagittal radius >10 km
Plane-elliptic	170 × 30 × 40	150 × 10	Major axis 2017.94, minor axis 64.137, eccentricity 1382.759, sagittal radius >10 km
Grating 1, 950 lines mm^−1^	170 × 40 × 40	150 × 20	Radius 35230, sagittal radius >10 km
Grating 2, 280 lines mm^−1^	170 × 40 × 40	150 × 20	Radius 34900, sagittal radius >10 km

## Data Availability

The shown experimental data are available at https://in.xfel.eu/metadata/doi/10.22003/XFEL.EU-DATA-004796-00.
